# Special issue on atomically controlled fabrication technology

**DOI:** 10.1186/1556-276X-9-232

**Published:** 2014-05-13

**Authors:** Yukio Takahashi, Yuji Kuwahara, Kazuto Yamauchi

**Affiliations:** 1 Department of Precision Science and Technology, Graduate School of Engineering, Osaka University, 2-1 Yamadaoka, Suita, Osaka 565-0871, Japan

## Editorial

The Global Center of Excellence (GCOE) for atomically controlled fabrication technology was established in 2008 by the Japanese Ministry of Education, Culture, Sports, Science and Technology (MEXT), as a succession program of the 21st Century COE program for atomistic fabrication technology promoted from 2003 to 2007. The GCOE program is implemented by three departments, namely the Departments of Precision Science & Technology, Applied Physics, and Advanced Science and Biotechnology, and by the Research Center for Ultra-precision Science and Technology, all of which belong to the Graduate School of Engineering of Osaka University.

The Fifth International Symposium on Atomically Controlled Fabrication Technology (ACFT-5) was organized by the GCOE program and the technical committee on ultraprecision machining of the Japan Society for Precision Engineering (JSPE), in cooperation with JSPE, the Japan Society of Applied Physics (JSAP), and the Physical Society of Japan (JPS).

The aim of our GCOE project is to achieve the atomic level controllability in wide-area processing and environmental harmony, which are essential for next-generation manufacturing technologies with high functions. For this purpose, by collaborating with other organizations from different fields, we focus not only on the creation of new fabrication processes beyond the current limitations but also on the systematization of the fabrication processes as science.

ACFT-5 highlights the recent achievements in the program. The topics covered here are ‘Impact of Quantum Beam Techniques with Advanced Optics’, ‘Surface Characterization and Bottom-up Technologies’, ‘Multi-Scale Surface Control Techniques’, ‘Advanced Processing and Materials Science for Device Applications’, and ‘Frontier in Carbon/Organic Nano-Scale Systems’. Finally, we would like to express our gratitude to all the contributors and committee members for their great effort in making the symposium successful and also to MEXT for its continuous support.

